# Angiotensin II type 2 receptor (AT2R) as a novel modulator of inflammation in rheumatoid arthritis synovium

**DOI:** 10.1038/s41598-017-13746-w

**Published:** 2017-10-16

**Authors:** Riccardo Terenzi, Mirko Manetti, Irene Rosa, Eloisa Romano, Felice Galluccio, Serena Guiducci, Lidia Ibba-Manneschi, Marco Matucci-Cerinic

**Affiliations:** 1 Department of Experimental and Clinical Medicine, Section of Internal Medicine, Rheumatology Unit, Azienda Ospedaliero-Universitaria Careggi (AOUC), University of Florence, Florence, Italy; 2 Department of Experimental and Clinical Medicine, Section of Anatomy and Histology, University of Florence, Florence, Italy

## Abstract

Despite increasing evidence suggesting that angiotensin II type 2 receptor (AT2R) may regulate tissue inflammation, no study has yet analyzed its possible implication in rheumatoid arthritis (RA) synovitis. In this study, we investigated the expression and function of AT2R in synovial tissue and cultured fibroblast-like synoviocytes (FLS) from RA patients. AT2R expression was strongly increased in RA compared with osteoarthritis (OA) synovium, as well as in in cultured RA-FLS respect to OA-FLS and healthy FLS. Treatment with pro-inflammatory cytokines was able not only to boost AT2R expression in RA-FLS and OA-FLS, but also to induce its *de novo* expression in healthy FLS. The stimulation of AT2R with the specific agonist CGP42112A significantly reduced gene expression of interleukin (IL)-1β and IL-6 and activation of NF-κB in RA-FLS, while opposite effects were elicited by AT2R small interfering RNA. Moreover, AT2R agonism efficiently decreased RA-FLS proliferation and migration either at baseline or under pro-inflammatory cytokine challenge. In conclusion, AT2R is strongly expressed in key effector cells of rheumatoid synovitis, namely RA-FLS, and the activation of AT2R with a specific agonist may effectively dampen their pro-inflammatory and aggressive behavior. AT2R agonism might represent a novel therapeutic strategy for patients with RA.

## Introduction

Rheumatoid arthritis (RA) is an autoimmune destructive disease of the joints characterized by chronic proliferative synovitis, infiltration of inflammatory cells into the synovial tissue, and articular cartilage destruction^1^. Articular damage is mainly driven by lymphocytes, macrophages and synovial lining fibroblasts, also called fibroblast-like synoviocytes (FLS) or type B synoviocytes^1^. FLS contribute to the initial stages of synovitis through the local production of pro-inflammatory cytokines, such as interleukin (IL)-1β, IL-6 and tumor necrosis factor (TNF)-α, and small-molecule mediators of inflammation^1,2^. Moreover, chronic articular exposure to pro-inflammatory cytokines confers to FLS a unique aggressive phenotype that can perpetuate joint destruction^2^. Indeed, RA-FLS share a number of features with transformed cells, including enhanced proliferation and production of proteolytic enzymes that degrade the extracellular matrix^1–3^. Hence, an important characteristic of the rheumatoid synovium is the marked hyperplasia of the lining layer, which is caused by an increased number of both FLS and macrophages^1–3^. However, the specific molecular mechanisms responsible for the hyperplasia and high activation state of RA-FLS remain to a large extent to be defined.

Nevertheless, RA is a systemic disease and extra-articular involvement is common. Patients with RA have a higher risk of mortality when compared with the general population, which is mainly due to increased cardiovascular disease related to both traditional risk factors and disease-induced chronic inflammation^4–6^. In this context, increasing evidence suggests that the renin-angiotensin system is dysregulated in RA^7^. Angiotensin II has two major G protein-coupled receptor subtypes, the angiotensin II type 1 receptor (AT1R) and the angiotensin II type 2 receptor (AT2R)^7^. It is well recognized that angiotensin II acts as a powerful pro-inflammatory mediator through the stimulation of AT1R and subsequent activation of NF-κB pathway^8,9^, contributing to the cardiovascular alterations of RA^7^. AT1R is highly expressed in cultured RA-FLS and in the hyperplastic synovium of rodent models of arthritis, where it has been proposed as a possible therapeutic target^10,11^. There is also evidence that angiotensin converting enzyme inhibitors or angiotensin II receptor blockers can ameliorate the clinical and laboratory parameters of RA^12–15^. While the implication of AT1R in the inflammatory process appears to be well defined, in such a context little is known about the contribution of AT2R. In fact, AT2R functions are still somewhat controversial, as it has been reported to either inhibit or promote inflammation in different experimental settings^8,16–25^. However, the majority of studies support the notion that AT1R and AT2R may mediate opposite effects, with AT2R mainly exerting an anti-inflammatory action^8,16–21^.

Despite the evidence that the renin-angiotensin system is involved in the pathogenesis of both articular and cardiovascular manifestations of RA and that AT2R may have a role in tissue inflammation, to the best of our knowledge no study has yet assessed the expression of AT2R in the chronically inflamed synovium of RA patients. On these bases, in the present study we investigated for the first time the expression of AT2R in synovial tissue and cultured FLS from patients with RA compared with osteoarthritis (OA). Moreover, we explored the potential of AT2R as a novel modulator of inflammation in the key effector cells of rheumatoid synovitis, namely RA-FLS.

## Results

### Expression of AT2R in RA and OA synovium

**Figure 1:**
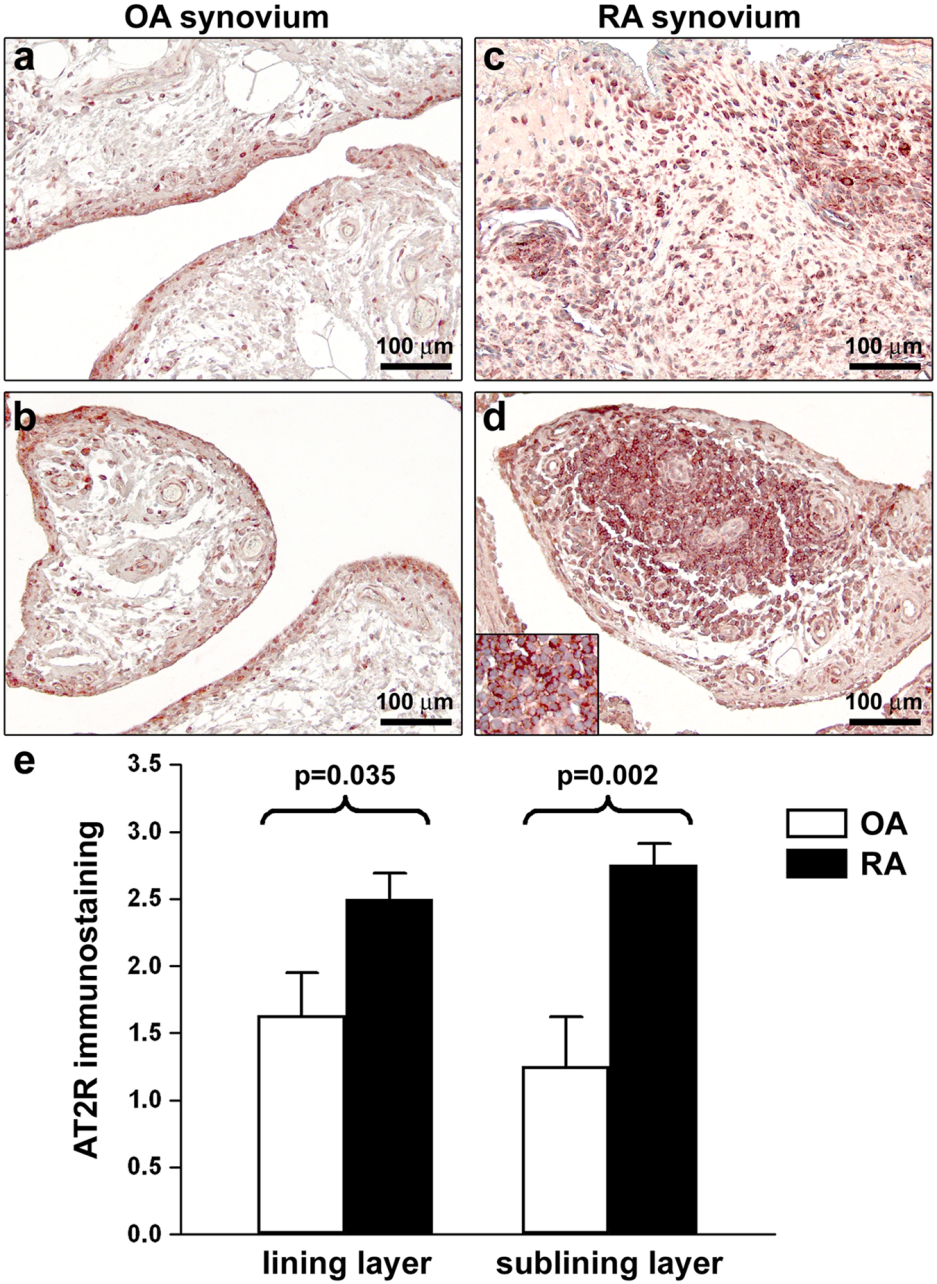
Expression of angiotensin II type 2 receptor (AT2R) in synovial tissue from patients with osteoarthritis (OA) and rheumatoid arthritis (RA). (**a–d**) Representative microphotographs of synovial sections from patients with OA (**a**,**b**) and patients with RA (**c**,**d**) immunostained with rabbit polyclonal anti-human AT2R antibody and counterstained with hematoxylin. AT2R immunostaining is stronger in cells of the hyperplastic RA synovial lining compared with OA synovial lining cells, as well as in RA sublining in respect to OA sublining (**a–c**). In RA synovium, ectopic lymphoid structures display a strong immunopositivity for AT2R (**d**). *Inset* in (**d**): higher magnification view of AT2R-immunopositive lymphocytes within an ectopic lymphoid structure of RA synovium. Scale bars are indicated in each panel. (**e**) Semiquantitative analysis of AT2R immunostaining in synovial lining and sublining layers. Data are mean ± SEM of immunostaining score performed on synovial sections from 8 patients with OA and 8 patients with RA. Student’s t-test was used for statistical analysis; p values are indicated.

Immunohistological analyses were carried out on synovial membrane sections from 8 patients with RA and 8 patients with OA. The expression of AT2R was detected either in the synovial lining layer or in the synovial sublining layer of all RA and OA specimens subjected to immunoperoxidase-based immunohistochemistry (Fig. 1a–d). AT2R immunostaining was stronger in cells of the hyperplastic RA synovial lining compared with OA synovial lining cells (Fig. 1a–c). Moreover, in RA synovial tissue ectopic lymphoid structures exhibited a strong immunopositivity for AT2R (Fig. 1d). As displayed in Fig. 1e, the analysis of immunostaining intensity confirmed that the expression of AT2R was significantly increased either in the lining or in the sublining layers of RA synovium compared with OA synovium (p = 0.035 and p = 0.002, respectively).

**Figure 2:**
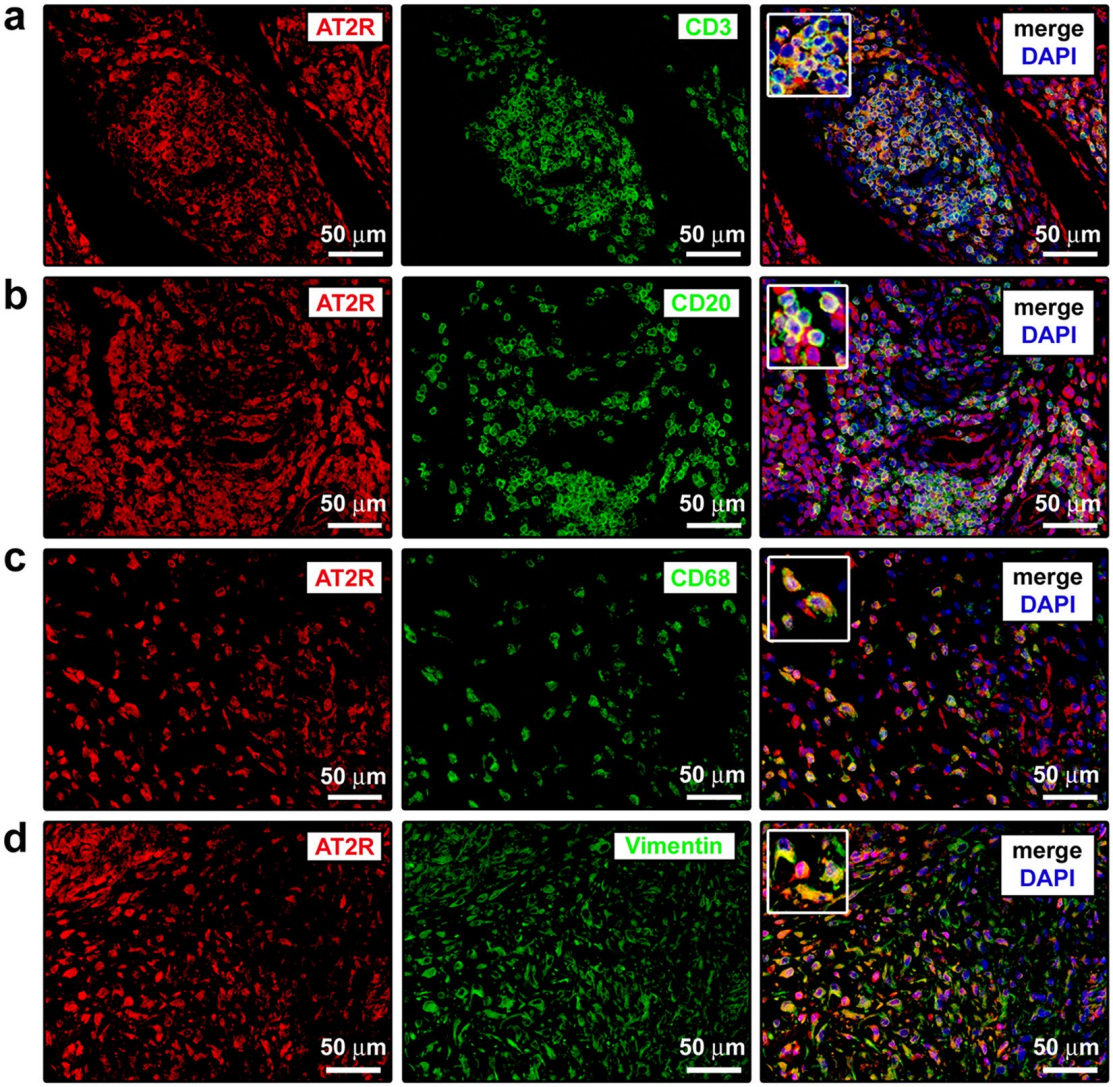
Expression of angiotensin II type 2 receptor (AT2R) in different cell types of rheumatoid arthritis (RA) synovium. (**a–d**) Representative microphotographs of synovial sections from patients with RA subjected to double immunofluorescence staining for AT2R (red) and the T cell marker CD3 (green) (**a**), the B cell marker CD20 (green) (**b**), the macrophage marker CD68 (green) (**c**) or the fibroblast marker vimentin (green) (**d**). Nuclei are counterstained with 4′,6-diamidino-2-phenylindole (DAPI; blue). Merge images are shown in the right panels. In RA synovium, AT2R is strongly expressed in CD3^+^ T cells (**a**) and CD20^+^ B cells (**b**) within ectopic lymphoid structures, CD68^+^ macrophages/type A synoviocytes (**c**) and vimentin^+^ fibroblast-like synoviocytes/type B synoviocytes (**d**). *Insets*: higher magnifications of double-immunopositive synovial cells from the respective panels. Scale bars are indicated in each panel.

We next examined the expression of AT2R in different cell types of RA synovium by double immunofluorescence staining combining anti-AT2R antibodies with antibodies raised against the T cell marker CD3, the B cell marker CD20, the macrophage marker CD68 or the fibroblast marker vimentin (Fig. 2a–d). As shown in Fig. 2a–d, in RA synovial tissue AT2R was strongly expressed in CD3^+^ T cells and CD20^+^ B cells within ectopic lymphoid structures, as well as in CD68^+^ macrophages (type A synoviocytes) and vimentin^+^ FLS (type B synoviocytes).

### AT2R expression and modulation by pro-inflammatory cytokines in cultured FLS from RA, OA and healthy synovium

**Figure 3:**
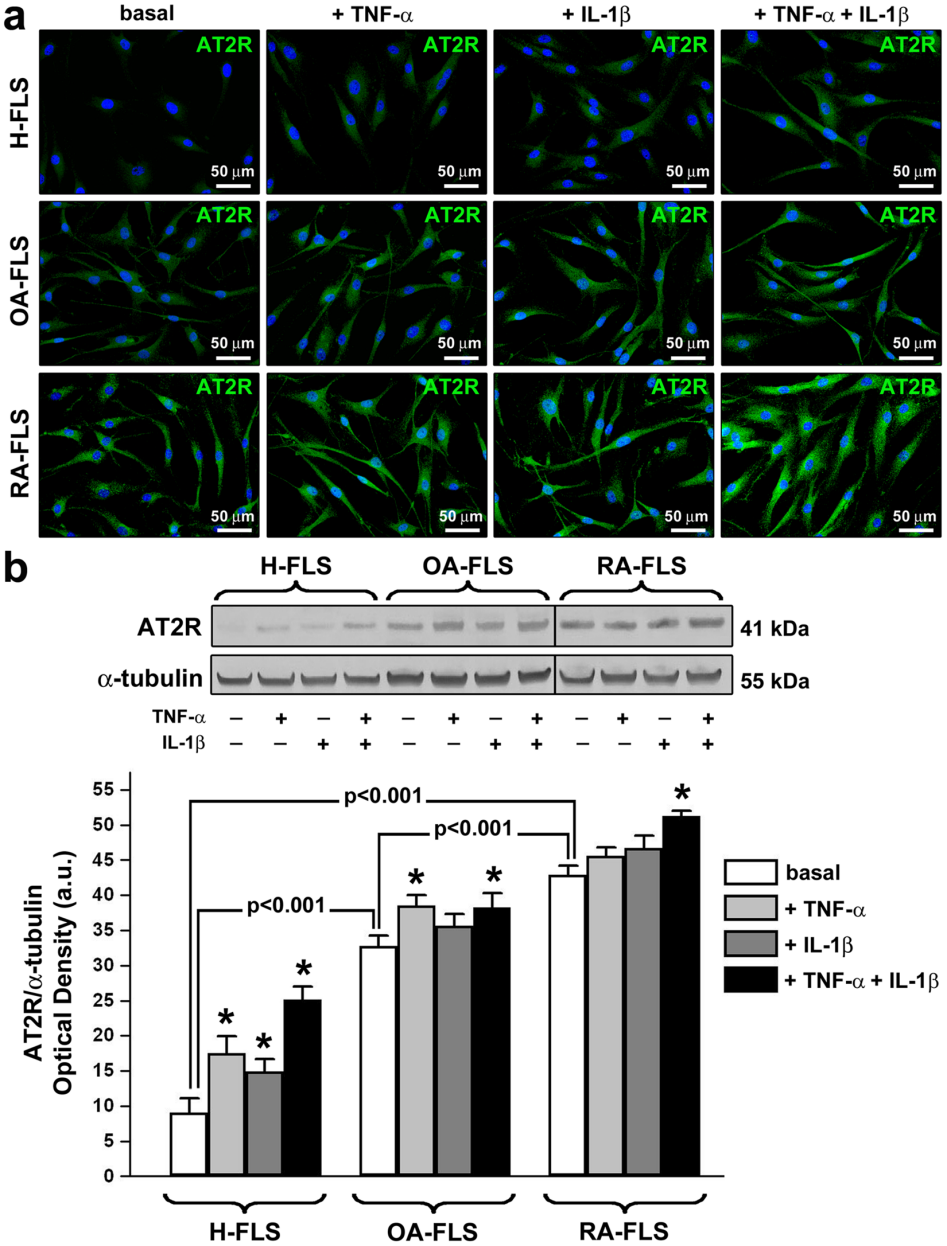
Expression of angiotensin II type 2 receptor (AT2R) in fibroblast-like synoviocytes (FLS) from healthy, osteoarthritis (OA) and rheumatoid arthritis (RA) synovium at baseline and after treatment with tumor necrosis factor (TNF)-α and interleukin (IL)-1β, alone or in combination. (**a**) Representative microphotographs of FLS isolated from healthy (H-FLS), OA (OA-FLS) and RA (RA-FLS) synovium subjected to immunofluorescence staining for AT2R (green) and nuclear counterstaining with 4′,6-diamidino-2-phenylindole (DAPI; blue). Scale bars are indicated in each panel. (**b**) Western blotting of total protein extracts from H-FLS, OA-FLS and RA-FLS at basal condition and treated with TNF-α and IL-1β, alone or in combination, assayed with anti-AT2R antibody; α-tubulin was used as a loading control. Representative cropped immunoblots are shown. All samples were run on the same gel. Non-adjacent samples were separated by a black line. Numbers on the right indicate molecular weight (kDa). A protein band with the expected molecular weight of 41 kDa was detected with the anti-AT2R antibody. The densitometric analysis of the bands normalized to α-tubulin is reported in the histograms. Data are mean ± SEM of optical density in arbitrary units (a.u.). Student’s t test was used for statistical analysis; *p < 0.05 *versus* the respective basal condition for each cell type. Results are representative of three independent experiments performed with each one of the six H-FLS, six OA-FLS and six RA-FLS lines.

The expression of AT2R in cultured FLS from healthy (H-FLS), OA (OA-FLS) and RA (RA-FLS) synovium was investigated by fluorescence immunocytochemistry and Western blotting at baseline and after treatment with the pro-inflammatory cytokines TNF-α and IL-1β, alone or in combination (Fig. 3a,b). H-FLS showed negligible basal expression of AT2R which was, instead, significantly increased after stimulation with TNF-α, IL-1β or combination of the two cytokines (p < 0.05 for all comparisons, Fig. 3a,b). Baseline expression of AT2R was significantly higher in OA-FLS than H-FLS (p < 0.001, Fig. 3a,b). Moreover, treatment of OA-FLS with TNF-α alone or in combination with IL-1β resulted in a significant increase in AT2R protein levels (p < 0.05 *versus* basal OA-FLS for both comparisons, Fig. 3a,b). At baseline, RA-FLS displayed the highest levels of AT2R (p < 0.001 *versus* basal H-FLS and OA-FLS, Fig. 3a,b). Stimulation with TNF-α and IL-1β further increased AT2R protein expression in RA-FLS resulting in significantly higher levels when cells were challenged with the two cytokines in combination (p < 0.05 *versus* basal RA-FLS, Fig. 3a,b).

### AT2R agonism reduces gene expression of pro-inflammatory cytokines and activation of NF-κB in RA-FLS

**Figure 4:**
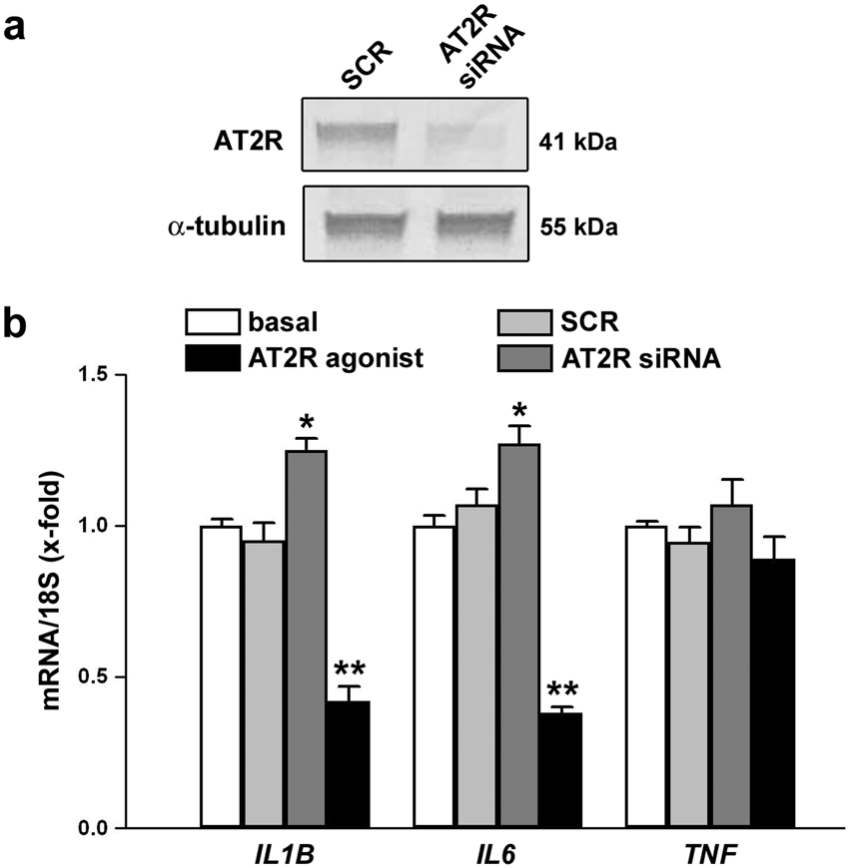
Effects of angiotensin II type 2 receptor (AT2R) gene silencing and AT2R activation with the agonist CGP42112A on the expression of pro-inflammatory cytokines in rheumatoid arthritis fibroblast-like synoviocytes (RA-FLS). (**a**) Verification of silencing capacity of AT2R small interfering RNA (siRNA). Protein levels of AT2R in RA-FLS transfected with AT2R siRNA or non-silencing scrambled RNA (SCR) were measured by Western blotting; α-tubulin was used as a loading control. Representative cropped immunoblots are shown. All samples were run on the same gel. Numbers on the right indicate molecular weight (kDa). (**b**) Gene expression of *IL1B*, *IL6* and *TNF* was evaluated by Real-Time PCR in RA-FLS at baseline, after transfection with AT2R siRNA or non-silencing SCR, and after treatment with AT2R agonist. The mRNA expression levels were normalized to the 18 S ribosomal RNA gene. For each gene, the mRNA expression in basal cells was set to 1; the other results are expressed as x-fold increase/decrease over basal response. Bars represent the mean ± SEM. Results are representative of three independent experiments performed with each one of the six RA-FLS lines. Statistical analysis was carried out with Student’s t test; *p < 0.05, **p < 0.001 *versus* the respective basal condition.

To clarify the possible function of AT2R in RA-FLS, cells were transfected with AT2R small interfering RNA (siRNA) or treated with the AT2R agonist CGP42112A and tested for mRNA expression of pro-inflammatory cytokines (Fig. 4a,b). Silencing of AT2R significantly increased the expression of *IL1B* and *IL6* genes (p < 0.05 *versus* basal condition), whereas *TNF* gene expression levels were not affected. As expected, transfection of RA-FLS with non-silencing scrambled RNA (SCR) did not modify the mRNA expression of the aforementioned cytokines compared with basal cells (Fig. 4b). In addition, activation of AT2R with AT2R agonist resulted in a significant reduction in gene expression of *IL1B* and *IL6* (p < 0.001 *versus* basal condition), but not of *TNF* (Fig. 4b).

**Figure 5:**
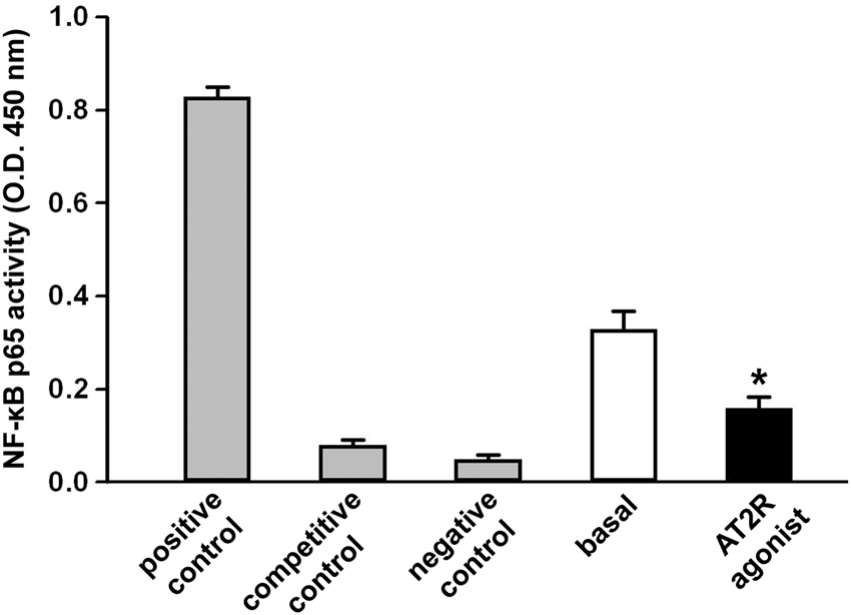
The angiotensin II type 2 receptor (AT2R) agonist CGP42112A reduces NF-κB p65 DNA binding activity in rheumatoid arthritis fibroblast-like synoviocytes (RA-FLS). NF-κB p65 DNA binding activity in RA-FLS at baseline and after treatment with AT2R agonist was determined by a NF-κB EZ-TFA transcription factor assay colorimetric kit. Positive, competitive, and negative controls were carried out using TNF-α-treated Hela whole cell extract, unlabeled competitor oligonucleotide containing the identical consensus sequence as the capture probe in other samples, and without any capture probe, respectively. Bars represent the mean ± SEM of optical density (O.D.) measured at 450 nm. Results are representative of three independent experiments performed with each one of the six RA-FLS lines. Statistical analysis was carried out with Student’s t test; *p < 0.05 *versus* basal condition.

Since NF-κB is a major transcription activator of the pro-inflammatory cytokine genes that were downregulated by AT2R agonism, we examined whether AT2R stimulation would directly affect NF-κB p65 DNA binding activity in RA-FLS. As displayed in Fig. 5, activation of AT2R with the AT2R agonist CGP42112A resulted in a significant reduction of NF-κB activity (p < 0.05 *versus* basal condition).

### AT2R agonism decreases RA-FLS proliferation and migration

**Figure 6:**
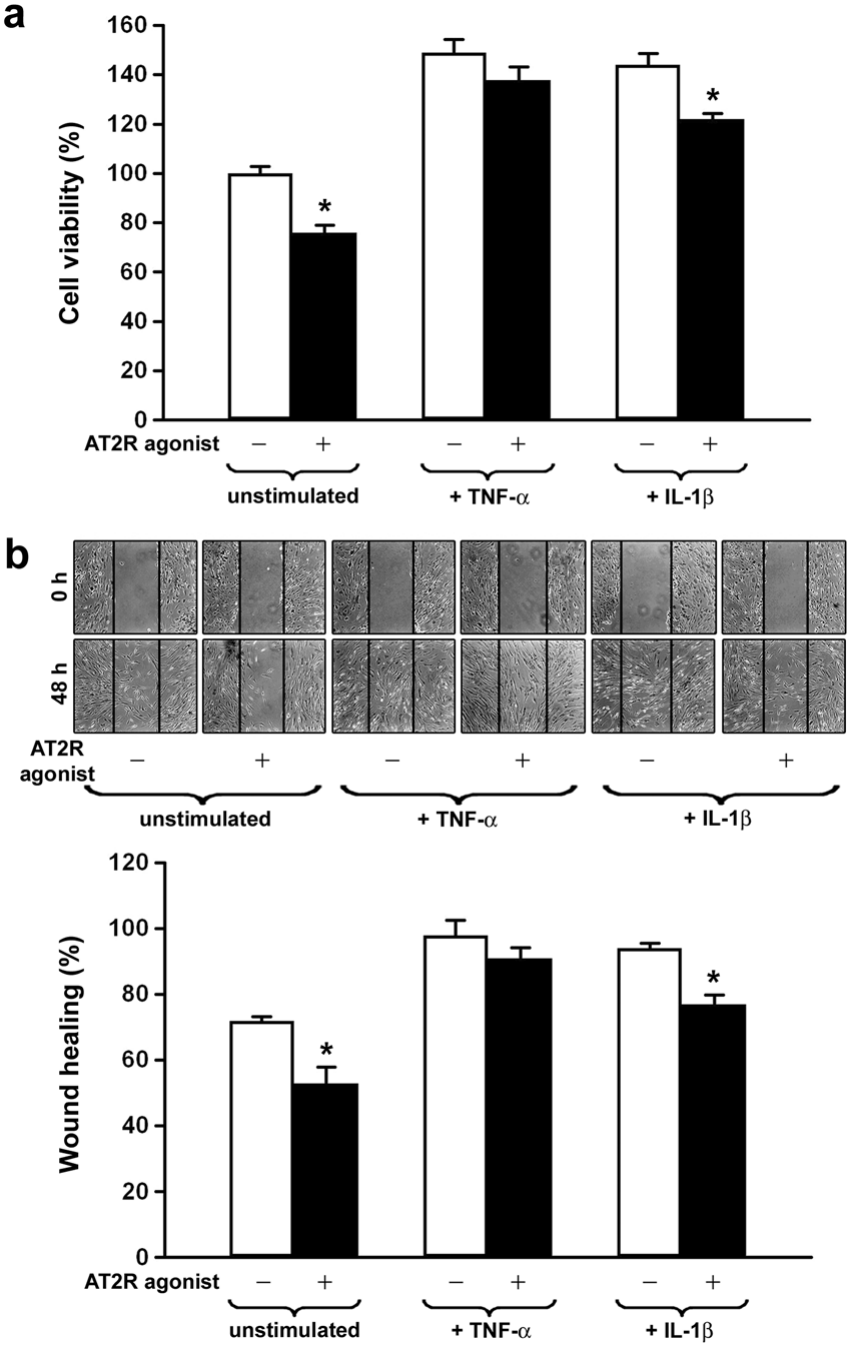
The angiotensin II type 2 receptor (AT2R) agonist CGP42112A decreases proliferation and migration of rheumatoid arthritis fibroblast-like synoviocytes (RA-FLS). (**a**) Cell viability evaluated at basal condition or after treatment with the pro-inflammatory cytokines tumor necrosis factor (TNF)-α and interleukin (IL)-1β in the presence/absence of AT2R agonist. Cell viability is expressed as the percentage increase/decrease over basal response (*i*.*e*. unstimulated cells without AT2R agonist). Bars represent the mean ± SEM. Results are representative of three independent experiments performed with each one of the six RA-FLS lines. Statistical analysis was carried out with Student’s t test; *p < 0.05 *versus* the respective condition without AT2R agonist. (**b**) Wound healing capacity of RA-FLS assayed at basal condition or after treatment with TNF-α and IL-1β in the presence/absence of AT2R agonist. A representative image of the wounded area at 0 hours and 48 hours after scratching is shown for each experimental condition (original magnification × 10). Histograms represent results of quantitative analysis of the percentage of wound repair. Data are mean ± SEM of three independent experiments performed with each one of the six RA-FLS lines. Statistical analysis was carried out with Student’s t test; *p < 0.05 *versus* the respective condition without AT2R agonist.

We evaluated the ability of the AT2R agonist CGP42112A to influence RA-FLS proliferation and migration either at baseline or after treatment with the pro-inflammatory cytokines TNF-α and IL-1β. To this aim, we carried out both WST-1 cell viability and wound healing assays. The addition of AT2R agonist to the culture medium significantly decreased cell viability and wound healing capacity either in unstimulated or in IL-1β-treated RA-FLS (p < 0.05 for all comparisons, Fig. 6a,b). A trend toward a reduction in cell viability and wound healing capacity was observed also when AT2R agonist was administered to TNF-α-treated RA-FLS, though these results did not reach statistical significance (Fig. 6a,b).

## Discussion

Our data demonstrate for the first time the presence of the AT2R receptor in human synovial tissue and in cultured human FLS. In particular, AT2R expression was observed in the synovial intimal lining and sublining layers from either patients with RA or patients with OA. Moreover, the expression of AT2R was different between RA and OA synovium. Indeed, all RA synovial specimens showed stronger immunostaining for AT2R than OA samples in cells of the lining and sublining layers. Of note, strong immunopositivity for AT2R was also detected in the ectopic lymphoid structures which were often found in RA synovial tissue. Double immunofluorescence staining further revealed that in RA synovium AT2R was strongly expressed in different cells of the adaptive and innate immune systems as well as in stromal cells which are known to drive the synovial inflammatory process, such as CD3^+^ T cells, CD20^+^ B cells, CD68^+^ macrophages and vimentin^+^ FLS. Collectively, these *ex vivo* findings suggest that AT2R expression may increase in a chronically inflamed microenvironment.

Interestingly, previous studies reported the presence of AT2R in the synovium of rats with adjuvant-induced arthritis as well as in cultured bovine chondrocytes stimulated with TNF-α or IL-1β pro-inflammatory cytokines, but not in unstimulated cells^26,27^. However, to the best of our knowledge, no *in vitro* study has previously investigated the expression and possible function of AT2R in FLS, which are considered leading players in the development of joint inflammation and destruction in RA^2^. Therefore, we herein analyzed the expression of AT2R in cultured FLS obtained from RA, OA and healthy synovium. In particular, we found high levels of AT2R in RA-FLS and OA-FLS at basal condition, while AT2R expression was negligible in basal H-FLS. Moreover, AT2R was more intensively expressed in RA-FLS compared with OA-FLS. Of note, treatment with TNF-α and IL-1β was able not only to foster the expression of AT2R in RA-FLS and OA-FLS, but also to induce its *de novo* expression in H-FLS. Thus, our *in vitro* findings clearly show that pro-inflammatory stimuli may induce and sustain the expression of AT2R in FLS. Consistent with these results, a large body of evidence supports the notion that in the adult organism AT2R is only sparsely expressed in healthy tissue, whereas receptor expression is strongly upregulated in case of tissue damage^28^. In particular, AT2R expression has been demonstrated in mesenchymal cells, including fibroblasts, in human tissues, especially in pathological and reparative processes^29–31^. Under these circumstances, it has been proposed that AT2R overexpression might provide a mechanism of restraining the cellular response to angiotensin II^30^. Indeed, based on its expression pattern, studies in transgenic and knockout animal models and cellular *in vitro* studies, AT2R appears to act mainly by counterbalancing the AT1R-mediated effects, such as those that have been linked to vascular, renal and cardiac fibroproliferative diseases^20,21,29,31–34^. Tissue-protective properties of AT2R in different conditions, including stroke, myocardial infarction, atherosclerosis, or neuronal damage, have been demonstrated in various *in vivo* studies in genetically altered animals or using selective receptor agonists and antagonists, with AT2R-mediated anti-inflammation being the predominant underlying molecular mechanism^35–38^.

As far as FLS are concerned, Pattacini *et al*.^11^ have previously shown that angiotensin II protects RA-FLS from apoptosis *via* the AT1R-dependent activation of the NF-κB pathway. However, the role of AT2R was not investigated in that study. In such a context, our findings about an increased expression of AT2R in RA-FLS prompted us to further elucidate the possible receptor implication in regulating the functional phenotype of these cells. We therefore investigated whether gain or loss of AT2R function could affect gene expression of pro-inflammatory cytokines and activation of NF-κB in RA-FLS. Our *in vitro* data demonstrated that activation of AT2R on RA-FLS with the selective agonist CGP42112A significantly downregulated the expression of *IL1B* and *IL6* genes, while opposite effects were observed when cells were silenced for AT2R. Moreover, treatment of RA-FLS with AT2R agonist significantly reduced the DNA binding activity of NF-κB p65, a well known transcriptional activator of the aforementioned pro-inflammatory genes. Consistent with our observations, Rompe *et al*.^19^ reported that AT2R stimulation reduced TNF-α-induced IL-6 levels *via* blocking NF-κB in primary human and murine dermal fibroblasts.

Since enhanced proliferation and migration of RA-FLS contribute substantially to synovial pannus growth and articular cartilage invasion^1–3^, we also tested the possible effects of AT2R agonism in WST-1 cell viability and wound healing experiments. Interestingly, we found that the stimulation of RA-FLS with the AT2R agonist CGP42112A significantly decreased cell viability and wound healing capacity. These findings are in agreement with previous evidence that AT2R may exert anti-proliferative and pro-apoptotic effects. In particular, in pathological conditions, such as heart failure, upregulation of AT2R inhibited angiotensin II-induced mitogen signals^30^. In AT2R transfected cells, overexpression of AT2R reduced proliferation mainly by counteracting AT1R signaling^39,40^. Moreover, expression of AT2R has been found to induce apoptosis in R3T3 fibroblasts or in different transfected cells even independently from angiotensin II stimulation^41^.

As proposed in different pathological states^28–31^, the upregulation of AT2R in the inflamed synovium of RA patients might be considered as an endogenous tissue protective system worth targeting for therapeutic purposes. Indeed, collectively our *in vitro* results indicate that the activation of AT2R with a specific agonist may effectively contribute to dampening of the pro-inflammatory and aggressive behavior of RA-FLS. This view is further corroborated by the experimental evidence that intra-articular injection of the AT2R agonist CGP42112A effectively decreased the severity of arthritis in a rat model of adjuvant-induced arthritis^26^. Of note, a recently developed selective non-peptide AT2R agonist, namely Compound 21, has demonstrated powerful anti-inflammatory properties, such as in ischemia/reperfusion injury, experimental atherosclerosis, renal inflammation and bleomycin-induced cutaneous inflammation^17,19–21,42^. Thus, the possible application of Compound 21 in the treatment of inflammation-related arthritis should be explored in future *in vitro* and *in vivo* studies. Since we reported that the overexpression of AT2R in the hyperplastic RA synovium is not restricted to FLS, it could be of interest to further investigate whether AT2R agonism may exhibit similar anti-inflammatory effects on other synovial cell types, such as macrophages/type A synoviocytes. In fact, it has been reported that AT2R stimulation is anti-inflammatory in lipopolysaccharide-activated macrophages through downregulation of TNF-α and IL-6 and increased production of IL-10^18^. Finally, we should consider that our study has some limitations due to the relatively small number of patient samples investigated. Therefore, future researches on larger cohorts of RA patients, possibly stratified according to disease stage and severity, are warranted.

In conclusion, our study demonstrated that (i) AT2R is overexpressed in the inflamed RA synovial tissue, (ii) the expression of this receptor is strikingly modulated in FLS by pro-inflammatory stimuli, and (iii) exogenous AT2R activation with a selective agonist might represent a potential novel therapeutic strategy for dampening of synovial inflammation in RA, though further translational studies will be required.

## Methods

### Synovial specimens

Synovial biopsies were collected from 8 patients with active RA^43^ and 8 age- and gender-matched patients with OA who underwent knee joint replacement. Each RA and OA synovial biopsy was divided into two specimens. One part was fixed in 10% buffered formalin and, after standard processing, embedded in paraffin and used for immunohistochemistry and microscopy analysis. The other part was used to obtain FLS. Patients had not received any disease-modifying antirheumatic drug treatment. FLS were also obtained from 6 age- and gender-matched healthy subjects who underwent post-traumatic knee surgical intervention. All subjects gave written informed consent, and the study protocol was carried out under the terms of the Declaration of Helsinki and approved by the local Institutional Review Board at the Azienda Ospedaliero-Universitaria Careggi, Florence, Italy.

### Cell isolation and culture

FLS were successfully isolated from RA (n = 6), OA (n = 6) and healthy (n = 6) synovial biopsies subjected to a mild proteolytic treatment with 0.05% trypsin and 0.5 mM EDTA in phosphate-buffered saline (PBS) for 10 minutes at 37 °C under gently shaking. Trypsin was then neutralized with fetal calf serum (FCS) (Celbio, Milan, Italy) and cells were plated in culture dishes (3 cm diameter, BD Falcon, BD Biosciences, San Jose, CA, USA) at a concentration of 1–1.5 × 10^6^ per dish in RPMI 1640 medium (Cambrex Bio Science, Milan, Italy) supplemented with 15% FCS, 2 mM glutamine and penicillin–streptomycin (Cambrex Bio Science). Once at confluence (about 3 weeks), the cell monolayers were expanded in culture by splitting 1:2 every 7 days. The cells were considered FLS (type B synoviocytes) if negative by immunostaining with anti-CD68, anti-CD14, anti-CD11b and anti-CD11c antibodies (markers of type A macrophage-like synoviocytes), positive by staining for the enzyme uridine diphosphoglucose dehydrogenase (preferentially expressed by the intimal lining FLS and reflecting their ability to synthesize hyaluronan, an important constituent of synovial fluid)^3^, and if they had a spindle-shaped, fibroblast-like morphology ( > 95% cell purity). Cell monolayers were used within the seventh passage in culture.

### Immunoperoxidase-based immunohistochemistry

After deparaffinization and rehydration, synovial tissue sections (5 µm thick) were boiled for 10 minutes in sodium citrate buffer (10 mM, pH 6.0) for antigen retrieval and treated with 3% H_2_O_2_ in methanol for 15 minutes at room temperature to block endogenous peroxidase activity. Sections were then incubated with Ultra V block (UltraVision Large Volume Detection System Anti-Polyvalent, HRP, catalog number TP-125-HL; Lab Vision, Fremont, CA, USA) for 10 minutes at room temperature. After blocking non-specific site binding, slides were incubated overnight at 4 °C with rabbit polyclonal anti-human AT2R antibody (1:250 dilution; catalog number ab19134; Abcam, Cambridge, UK) diluted in 1% bovine serum albumin (BSA) in PBS. The day after, tissue sections were washed in PBS and incubated with biotinylated secondary antibodies (Lab Vision) for 10 minutes at room temperature. Subsequently, the slides were washed in PBS and incubated with streptavidin peroxidase (Lab Vision) for 10 minutes at room temperature. Immunoreactivity was developed using 3-amino-9-ethylcarbazole (AEC kit, catalog number TA-125-SA; Lab Vision) as chromogen. Synovial sections were finally counterstained with hematoxylin and observed under a Leica DM4000 B microscope (Leica Microsystems, Mannheim, Germany). Sections not exposed to primary antibodies or incubated with isotype-matched and concentration-matched non-immune rabbit IgG (Sigma-Aldrich, St. Louis, MO, USA) were included as negative controls for antibody specificity. Light microscopy images were captured with a Leica DFC310 FX 1.4-megapixel digital colour camera equipped with the Leica software application suite LAS V3.8 (Leica Microsystems). AT2R immunostaining was quantified in a semiquantitative manner, where (0) indicates no staining, (1) weak staining, (2) moderate staining and (3) strong staining in cells of the lining and sublining layers at eight randomly chosen high-power fields (40 × original magnification) per sample. Two different examiners performed the evaluation blindly. When there was interobserver disagreement, the specimen was reviewed again by both observers and the disagreement resolved.

### Fluorescence immunohistochemistry

Synovial tissue sections (5 µm thick) were deparaffinized, rehydrated and boiled for 10 minutes in sodium citrate buffer (10 mM, pH 6.0). Sections were incubated in 2 mg/ml glycine for 10 minutes to quench autofluorescence and then blocked for 1 hour at room temperature with 1% BSA in PBS. The sections were subsequently incubated overnight at 4 °C with the following primary antibodies diluted in PBS with 1% BSA: rabbit polyclonal anti-human AT2R (1:250 dilution; catalog number ab19134; Abcam), mouse monoclonal anti-human CD3 (1:10 dilution; catalog number ab17143; Abcam), mouse monoclonal anti-human CD20 (1:200 dilution; catalog number M0755; Dako, Glostrup, Denmark), mouse monoclonal anti-human CD68 (1:50 dilution; catalog number M0876; Dako) and mouse monoclonal anti-human vimentin (1:50 dilution; catalog number M7020; Dako). The day after, the slides were washed in PBS and incubated for 45 minutes at room temperature in the dark with Alexa Fluor-488 conjugated goat anti-mouse IgG or Rhodamine Red-X-conjugated goat anti-rabbit IgG (Invitrogen, San Diego, CA, USA) diluted 1:200 in PBS with 1% BSA, as secondary antibodies. Double immunofluorescence staining was performed by mixing mouse and rabbit primary antibodies and subsequently mixing fluorochrome-conjugated secondary antibodies. Irrelevant isotype-matched and concentration-matched mouse and rabbit IgG (Sigma-Aldrich) were used as negative controls. Nuclei were counterstained with 4′,6-diamidino-2-phenylindole (DAPI; Chemicon International, Temecula, CA, USA). Synovial sections were then examined with a Leica DM4000 B microscope equipped with fully automated fluorescence axes (Leica Microsystems). Fluorescence images were captured with a Leica DFC310 FX 1.4-megapixel digital colour camera (Leica Microsystems).

### Fluorescence immunocytochemistry

FLS were seeded onto glass coverslips, grown to 70% confluence and serum-starved for 24 hours. In some experimental points, FLS were subsequently incubated for 24 hours in RPMI 1640 medium with 0.2% FCS containing TNF-α (0.1 ng/ml; PeproTech, Rocky Hill, NJ, USA) and IL-1β (15 pg/ml; PeproTech), alone or in combination. At the end of the experiments, cells were fixed with 3.7% buffered paraformaldehyde and permeabilized with 0.1% Triton X-100 in PBS. Slides were washed with PBS and blocked with 1% BSA in PBS for 1 hour at room temperature, and were then incubated overnight at 4 °C with rabbit polyclonal anti-human AT2R antibody (catalog number ab19134; Abcam) at 1:500 dilution in 1% BSA in PBS, followed by incubation for 45 minutes at room temperature in the dark with Alexa Fluor-488-conjugated secondary antibodies at 1:200 dilution (Invitrogen). Irrelevant isotype-matched and concentration-matched rabbit IgG (Sigma-Aldrich) were used as negative controls. Nuclei were counterstained with DAPI. Immunolabeled cells were examined with a Leica DM4000 B microscope (Leica Microsystems) and fluorescence images were captured with a Leica DFC310 FX 1.4-megapixel digital colour camera (Leica Microsystems).

### Western blotting

Proteins were extracted from FLS at basal conditions or after treatment for 24 hours with TNF-α (0.1 ng/ml; PeproTech) and IL-1β (15 pg/ml; PeproTech), alone or in combination. FLS were lysed in ice-cold lysis buffer (10 mM Tris HCl, pH 7.4, 150 mM NaCl, 1% Triton X-100, 0.25% sodium dodecyl sulphate) supplemented with 1 mM sodium orthovanadate, 1 mM NaF, 1 mM EDTA, 1 mM phenylmethylsulphonyl fluoride, and 10 µg/ml aprotinin. The solution was cleared by centrifugation and assayed for protein content using Bradford’s method. Twenty micrograms of total proteins were electrophoresed on NuPAGE 4 to 12% Bis-Tris Gel (Invitrogen) and blotted onto polyvinylidene difluoride membranes (Invitrogen). The membranes were blocked with blocking solution included in the Western Breeze Chromogenic Western Blot Immunodetection Kit (Invitrogen) for 30 minutes at room temperature on a rotary shaker and then incubated for 1 hour at room temperature with rabbit polyclonal anti-human AT2R (1:1,000 dilution; catalog number ab19134; Abcam) and rabbit polyclonal anti-α-tubulin (1:1,000 dilution; catalog number #2144; Cell Signaling Technology, Danvers, MA, USA) antibodies, assuming α-tubulin as control invariant protein. Immunodetection was performed as described in the Western Breeze Chromogenic Immunodetection protocol (Invitrogen). Densitometric analysis of the bands was performed using ImageJ software (NIH, Bethesda, Maryland, USA; online at http://rsbweb.nih.gov/ij) and the values were normalized to α-tubulin.

### Gene silencing of AT2R

RA-FLS were seeded shortly before transfection. The cells were transfected for 48 hours with 10 nM of AT2R siRNA (AT2 siRNA; catalog number sc-29752; Santa Cruz Biotechnology, Dallas, Texas, USA) or non-silencing SCR (Control siRNA-A; catalog number sc-37007; Santa Cruz Biotechnology) using HiPerFect transfection reagent (Qiagen, Milan, Italy) according to the manufacturer’s instructions.

### RNA isolation and quantitative Real-Time PCR

Total RNA was isolated from RA-FLS at basal conditions, after transfection with AT2R siRNA or non-silencing SCR or after treatment for 24 hours with the AT2R agonist CGP42112A (10^−5^ M) (catalog number C160; Sigma-Aldrich) using the RNeasy Micro Kit (Qiagen). First strand cDNA was synthesized using the QuantiTect Reverse Transcription kit (Qiagen). For mRNA quantification, SYBR Green Real-Time PCR was performed using the StepOnePlus Real-Time PCR System (Applied Biosystems, Milan, Italy) with melting curve analysis. Predesigned oligonucleotide primer pairs were obtained from Qiagen (QuantiTect Primer Assay). The assay IDs were Hs_IL1B_1_SG (human IL-1β; catalog number QT00021385), Hs_IL6_1_SG (human IL-6; catalog number QT00083720), Hs_TNF_1_SG (human TNF-α; catalog number QT00029162), and Hs_RRN18S_1_SG (human 18 S rRNA; catalog number QT00199367). Amplification was performed according to a standard protocol recommended by the manufacturer. 18S ribosomal RNA was measured as an endogenous control to normalize for the amounts of loaded cDNA. Differences were calculated with the threshold cycle (Ct) and comparative Ct method for relative quantification. All measurements were performed in triplicate.

### Detection of NF-κB p65 DNA binding activity

Nuclear proteins of RA-FLS at basal conditions or after treatment for 24 hours with the AT2R agonist CGP42112A (10^−5^ M) were isolated by using the CelLytic NuCLEAR Extraction Kit (catalog number NXTRACT; Sigma-Aldrich) according to the manufacturer’s instructions. Protein concentration was determined by BCA Protein Assay Kit (Bio-Rad Laboratories, Hercules, CA, USA) and 50 μg nuclear proteins were used to measure the NF-κB p65 DNA binding activity using a commercial NF-κB EZ-TFA transcription factor assay colorimetric kit (catalog number 70–520; Merck Millipore, Billerica, MA, USA) according to the manufacturer’s protocol. Positive, competitive, and negative controls were carried out using TNF-α-treated Hela whole cell extract, unlabeled competitor oligonucleotide containing the identical consensus sequence as the capture probe in other samples, and without any capture probe, respectively.

### Cell viability assay

RA-FLS were seeded onto 96-well plates (20 × 10^3^ cells per well) in RPMI 1640 with 15% FCS and were left to adhere overnight. Cells were then washed three times with serum-free medium and incubated in RPMI 1640 with 0.2% FCS for an additional 24 hours. Then, FLS were incubated for 24 hours in RPMI 1640 with 0.2% FCS containing TNF-α (0.1 ng/ml) or IL-1β (15 pg/ml), alone or in combination with the AT2R agonist CGP42112A (10^–5^ M). Cell viability was determined by a cell proliferation assay using the WST-1 (4-[3-(4-iodophenyl)-2-(4-nitrophenyl)-2H-5-tetrazolio]-1,3-benzene disulfonate) reagent (Roche Diagnostics, Mannheim, Germany) according to the manufacturer’s instructions. All measurements were performed in triplicate and the results were expressed as the percentage increase/decrease in cell viability over basal response.

### Wound healing assay

RA-FLS were cultured in 24-well plates at a density of 2 × 10^5^ cells/ml; 90% confluence allowed one parallel ‘wound’ in each well, created in the centre of each well by scratching with a sterile P200 pipette tip. Serum-free medium was used to remove the debris. Cells were then incubated in RPMI 1640 with 0.2% FCS containing TNF-α (0.1 ng/ml) or IL-1β (15 pg/ml), alone or in combination with the AT2R agonist CGP42112A (10^−5^ M). All experimental conditions were performed in triplicate. The wounded area was observed at 0, 24 and 48 hours after scratching and photographed under a light inverted microscope (Leica Microsystems) equipped with a digital camera. At 0 and 48 hours after scratching, measurements of wound area (*i*.*e*. area not occupied by cells) were made on digitized images at three randomly chosen points from each well using ImageJ software (NIH). The extent of wound closure was presented as the percentage by which the original scratch area had decreased at 48 hours.

### Statistical analysis

Statistical analyses were performed using the Statistical Package for Social Sciences (SPSS) software for Windows, version 20.0 (SPSS, Chicago, IL, USA). Data are expressed as mean ± standard error of the mean (SEM). Data normality was tested using the Shapiro-Wilks test. The Student’s t-test was used for statistical evaluation of the differences between two independent groups. A p value < 0.05 according to a two-tailed distribution was considered statistically significant.

### Data availability

All relevant data are within the paper.
